# Modeling of slip rate-dependent traversability for path planning of wheeled mobile robot in sandy terrain

**DOI:** 10.3389/frobt.2024.1320261

**Published:** 2024-01-25

**Authors:** Go Sakayori, Genya Ishigami

**Affiliations:** Graduate School of Integrated Design Engineering, Faculty of Science and Technology, Keio University, Tokyo, Japan

**Keywords:** wheel-soil interaction, rough terrain, slip rate, slip ratio, terramechanics, path planning

## Abstract

A planetary exploration rover has been employed for scientific endeavors or as a precursor for upcoming manned missions. Predicting rover traversability from its wheel slip ensures safe and efficient autonomous operations of rovers on deformable planetary surfaces; path planning algorithms that reduce slips by considering wheel-soil interaction or terrain data can minimize the risk of the rover becoming immobilized. Understanding wheel-soil interaction in transient states is vital for developing a more precise slip ratio prediction model, while path planning in the past assumes that slips generated at the path is a series of slip ratio in steady state. In this paper, we focus on the transient slip, or slip rate the time derivative of slip ratio, to explicitly address it into the cost function of path planning algorithm. We elaborated a regression model that takes slip rate and traction force as inputs and outputs slip ratio, which is employed in the cost function to minimize the rover slip in path planning phase. Experiments using a single wheel testbed revealed that even with the same wheel traction force, the slip ratio varies with different slip rates; we confirmed that the smaller the absolute value of the slip rate, the larger the slip ratio for the same traction force. The statistical analysis of the regression model confirms that the model can estimate the slip ratio within an accuracy of 85% in average. The path planning simulation with the regression model confirmed a reduction of 58% slip experienced by the rover when driving through rough terrain environments. The dynamics simulation results insisted that the proposed method can reduce the slip rate in rough terrain environments.

## 1 Introduction

Enhancing the autonomy of planetary rovers stands as a critical need in the exploration of expansive celestial surfaces within tight mission schedules. An illustrative instance is the NASA Mars Sample Return campaign’s demand for the sample fetch rover to cover distances of up to 20 km within 150 Martian solar days, aimed at gathering a variety of materials, including rocks, soils, and atmospheric data (Muirhead et al., 2020). Historically, Martian rovers equipped with autonomous mobility faced limitations in achieving swift traversal due to frequent human interventions necessitated by the evaluation of potentially hazardous terrain in the presence of communication delays. Additionally, rover slippage on extraterrestrial terrains, particularly deformable surfaces, has proven to be a significant concern, as illustrated by the significant slippage experienced by the Curiosity rover on the rippled sand within Hidden Valley (Rankin et al., 2020). This necessitated adjustments to the route for safer paths. Therefore, it is crucial to conduct a dependable assessment of terrain traversability on deformable surfaces, as this enables faster and more extensive rover exploration.

When a wheeled-based rover traverses on loose soil, it experiences complex interaction between the wheel and soil, including soil deformation, particle displacement, and slip. The occurrence of large slip while traveling can notably impact the rover’s traction, steering, and overall mobility, resulting in increased power consumption and reduced operational efficiency. Therefore, revealing the mechanisms of the wheel-soil interaction and optimizing the rover motion is crucial for developing reliable planetary rovers. To pre-optimize the rover motion, path planning algorithms considering the wheel-soil interaction would be essential.

Most of the work related to rover path planning addresses steady state motion, while few works consider transient state motion. Here, a *steady state* refers to a situation where both the velocity command to the rover and its translational velocity are constant. This is a commonly observed state when traveling on flat terrains. On the other hand, a *transient state* refers to a situation where either the velocity command value, the actual translational velocity, or both are changing over time. For instance, at the start of traveling on a slope, even if the velocity command remains constant, the translational velocity can change due to the influence of gravitational factors. There are also cases, such as during braking, where the velocity command changes, but the translational velocity remains constant. Velocity change causes acceleration, therefore, translational acceleration or acceleration command occurs in transient states. During planetary exploration, the rover is likely to experience transient state motion. Therefore, understanding the wheel-soil interaction under varying acceleration condition is crucial for optimizing rover motion.

In this study, we aim to investigate the importance of the acceleration dimension in the wheel-soil interaction and its impact on the path planning phase for planetary rovers. First, we introduce the concept of “*slip rate*”, which is the time derivative of the slip ratio, and reveal how the forces acting on the wheel change when it travels at a constant slip rate. Here, slip rate is a physical quantity that considers both the angular acceleration and translational acceleration of the wheel. Subsequently, we clarify the impact of slip rate on the relationship between traction force and slip ratio and develop a regression model. Using this relationship, we apply it to aid the path planning algorithm for planetary rovers. Lasetly, simulation studies confirmed that it is possible to reduce the slip ratio experienced by the rover when driving on rough terrains.

The contributions of this paper are as follows:

• We defined the term *slip rate*, and explain the physical phenomena.
• We conducted single-wheel experiments under constant slip rate conditions and revealed that the same traction force can generate different slip ratios depending on the slip rate.
• We designed regression models for traction force *versus* slip ratio that are dependent on the slip rate, to be employed in the path planning cost function.

The rest of the paper is organized as follows: Section 2 introduces related research and reveals the issues when considering transition state motion. Section 3 shows the results of the slip rate experiment obtained from the single wheel testbed and discusses the effect of slip rate. Section 4 explains the path planning method employing a regression model that takes slip rate, traction force as the input and outputs slip ratio. Section 5 draws the conclusion and presents a direction for future studies.

## 2 Related research

This paper integrates the notion of wheel-soil interaction, and path planning considering traversability and slip risks. The following subsections summarize the related literature in these fields.

### 2.1 Terramechanics

A classical wheel-soil interaction model based on terramechanics has been widely employed for mobility analysis of wheeled robots and utilized for wheel traction control to reduce the risks of wheel slip. Recent research has developed a wheel-soil interaction model for a lightweight vehicle based on comprehensive measurements of the wheel driving characteristics on loose soil (Horiko and Ishigami, 2020; Tsubaki and Ishigami, 2021). This model can provide insights into the complex physics of the wheel-soil interaction and help optimize the design and control of wheel-based rovers for various terrain conditions. However, due to the complex shape of the contact surface between the wheel and the soil, the distribution of shear stresses and normal stresses greatly depends on the wheel shape, making it challenging to discuss uniformly. Moreover, computing net traction force or rolling resistance using simple integral calculations is also difficult. A modeling method to address such problems is the Resistive Force Theory (RFT) (Li et al., 2013; Agarwal et al., 2019). The feature of RFT is finding consistency in the behavior of soil and enabling forces to be calculated with a single parameter. Dynamic Resistive Force Theory (DRFT) has been developed as a modeling method suitable for wheels traveling at high speeds, by extending RFT with the introduction of velocity dependency (Agarwal et al., 2021).

However, an open issue is that none of the wheel-soil interaction models reveal the mechanical phenomena in the transient state. Incorporating a time dependent term into the terramechanics equation or wheel-soil models that take acceleration dimensions into account will be indispensable to improve the prediction accuracy of rover slip.

### 2.2 Terrain traversability prediction

Numerous research studies have been proposed to estimate the slip ratio by utilizing information obtained from sensors mounted on wheels and rovers (Cross et al., 2013; Bouguelia et al., 2017; Song et al., 2017; Kim and Cho, 2023). Omura et al. (Omura and Ishigami, 2017) proposed a machine learning algorithm, which classifies the wheel slippage into three categories: non-stuck wheel, quasi-stuck wheel, and stuck wheel. The force applied to the wheel and the contact range information are taken as inputs to output the slippage category. Gonzalez et al. (2018) proposed an unsupervised learning model to detect various levels of slip and also explored the optimal placement of IMU sensors on the rover chassis to optimize slip detection.

Various planning algorithms are proposed to predict terrain traversability utilizing visual and geometric information. Camera images are employed to classify the terrain and to predict the slippage of each terrain (Helmick et al., 2009; Brooks and Iagnemma, 2012; Ono et al., 2015; Otsu et al., 2016; Rothrock et al., 2016; Feng et al., 2022; Wang et al., 2023). Regression-based algorithms model traversability in accordance with wheel slip, which is often estimated from terrain inclination (Skonieczny et al., 2019). Cunningham et al. (2017) trained a machine learning algorithm to predict slip from both terrain type and slope using data from the Curiosity rover. Hedrick et al. (2020) proposed online map updates with information gathered around the rover.

However, the prediction models determine only the feasibility/infeasibility of driving in a steady state, and they do not address predictions in the transient state. Since the traversable area should differ depending on the driving conditions, it is necessary to understand the phenomena of slip in transient state and to create slip prediction model including transient state phenomena. Modeling the slip prediction, it could be used in the path planning phase.

### 2.3 Risk aware planning

Numerous risk-aware planning methods have been proposed in which mobility risk is expressed as a function of rover wheel slippage, with the generation of paths that constrain risk (Lee et al., 2016; Endo et al., 2023; Park and Chung, 2023). Inotsume et al., (2020) proposed a path planning algorithm based on Rapidly-exploring Random Trees (RRT), allowing users to define slip-based risk thresholds. Candela and Wettergreen (2022) proposed a probabilistic path planning framework that quantifies science investigation and mobility risk of rover exploration, where they employed the slip prediction model proposed by Cunningham et al. (2017). Mizuno and Kubota (2020) also use a RRT* based path planner, but they model slip uncertainty with particle filters. Endo and Ishigami (2022) propose a framework that updates the latent traversability model by exploring informative terrain under the constraints of stochastic rover slip.

Risk-aware planning considers the slip ratio as one type of risk, but it was a series of steady-state slip. Understanding the phenomena of slip in transient state will enable to predict risks that are close to natural phenomena.

## 3 Slip rate experiment

This section describes the definition of slip rate, and discusses the result obtained from the constant slip rate experiment. Section 3.1 defines the term slip rate and explains expected physical phenomena of wheels. Section 3.2 explains the experimental setup and condition to conduct a constant slip rate experiment. Section 3.3 discusses the result obtained from constant slip rate experiment.

### 3.1 Definition of slip rate

The longitudinal slip *s*, defined as the ratio between the wheel’s circumferential velocity *rω* and the translational velocity *v*_*x*_, is defined as the following equation:

$$s=\left\{\begin{array}{cc} 1-\frac{v_{x}}{r\omega }\; & \left(v_{x}\leq r\omega \right)\\ \frac{r\omega }{v_{x}}-1\; & \left(v_{x}>r\omega \right) \end{array}\right.$$ (1)

where *r* is the wheel radius and *ω* is the angular velocity of the wheel. Negative slip, a rare phenomenon, occurs in specific situations such as when braking is applied but the rover continues to descend a slope. Therefore, this paper will only address regions where positive slip occurs.

The time derivative of the longitudinal slip $\dot{s}$ (hereafter referred to as the slip rate) can be written as follows:

$$\dot{s}=\frac{rv_{x}\dot{\omega }-r\omega {\dot{v}}_{x}}{r^{2}\omega^{2}}\;\left(v_{x}\leq r\omega \right)$$ (2)

When the rover is controlled with a target of a constant speed, the wheel is moving at a constant circumferential velocity; it maintains a constant rotational angular velocity. Under this condition, the slip ratio can be described as follows:

$$\dot{s}=\frac{-{\dot{v}}_{x}}{r\omega }\;\left(∵\;\dot{\omega }=0\right)$$ (3)

Depending on the sign of the time derivative of the translational velocity, which is the translational acceleration, the sign of the slip rate is determined. The value of the slip rate is determined by the ratio of translational acceleration to circumferential velocity. Specifically, a positive slip rate indicates a state where the rover is approaching a stuck condition, whereas a negative slip rate signifies a state where it is escaping from stuck. The value of the slip rate signifies the speed at which it becomes stuck or escaping from stuck.

When the rover adjusts its translational velocity via accelerating, the sign and value of the slip rate are determined based on the relationship between the wheel’s rotational angular velocity, rotational angular acceleration, translational velocity, and translational acceleration. When the translational velocity is constant due to external factors, such as during a slope ascent, the slip ratio at that instant can be calculated from the ratio of angular velocity to angular acceleration, as described in Eq. 4.

$$\dot{s}=\frac{v_{x}\dot{\omega }}{r\omega^{2}}=\left(1-s\right)\frac{\dot{\omega }}{\omega }\;\left(∵{\dot{\;v}}_{x}=0\right)$$ (4)

In this study, to reveal the differences in wheel-soil interaction, the slip rate was set as a variable under conditions of constant circumferential velocity, considering the actual operation of planetary rovers. The experimental setup will be discussed in the following subsection.

### 3.2 Experimental setup

**FIGURE 1:**
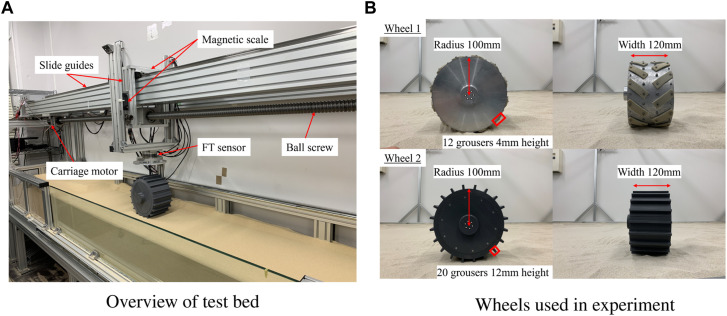
Experimental setup. **(A)** Overview of test bed. **(B)** Wheels used in experiment.

The single wheel test bed, as shown in Figure 1A, is filled with soil with a dimension of 3,500 mm in length, 600 mm in width, and 1,200 mm in depth. The experimental setup comprises a carriage stage equipped with a wheel that possesses the capability to move both horizontally and vertically through the utilization of slide guides. The stage is further linked to a carriage motor via a timing pulley belt. Therefore, this test bed possesses the capability to adjust the wheel’s circumferential velocity *rω* and translational velocity *v*_*x*_ relative to the ground; the wheel slip can be arbitrarily adjusted. The time derivative of the horizontal displacement of the carriage measured by the magnetic scale (linear encoder) on the wheel test bed provides *v*_*x*_, and the motor encoder measures the wheel angle of rotation with respect to time, giving the angular velocity *ω*. Feedback controls are performed for the wheel motor and the carriage motor to obtain the desired circumferential velocity *rω* and translational velocity *v*_*x*_. The vertical displacement measured by the magnetic scale on the wheel test bed provides the sinkage of the wheel. To measure the forces and torques applied to the entire wheel, we use a 6-axis force/torque sensor. The force measured in the longitudinal direction by this sensor is the net traction force (hereinafter traction force).

In this paper, to maintain a constant slip rate, we decided to keep the circumferential velocity constant and vary the translational velocity; by adjusting the translational acceleration we can achieve a constant slip rate. The angular velocity *ω* was set to 10° and 20°/s to achieve constant circumferential velocity, taking into consideration that the selected values align with the operational context of the operated rovers. The initial slip ratio was set to 0.0, then the carriage motor moved the wheel with slip rates of 1, 2, 5, 10, and 20 1/s to simulate scenarios where the wheel is gradually becoming stuck (*s* = 1.0). On the other hand, to simulate scenarios where the wheel is escaping from stuck, we set the initial slip ratio to 1.0 and employed slip rates of −1, −2, −5, −10, and −20 1/s. Additionally, to simulate situations where the wheel gets stuck and then escapes, we started with an initial slip ratio of 0.0, moved the wheel to 1.0 slip ratio with a constant slip rate, and then moved it back to 0 slip ratio with a negative constant slip rate (absolute value of the slip rate is equivalent). In this paper, we call this experimental condition a *round trip*. Two types of wheels with grousers, as shown in Figure 1B, were adopted to reveal the relationship between traction force and slip ratio. For each wheel, by loading an appropriate weight on the top of the wheel, we achieved a condition where a load of 10 kg is applied to the wheel. The soil covering the wheel test bed is Toyoura sand (Zhang et al., 2013).

### 3.3 Experimental results

**FIGURE 2:**
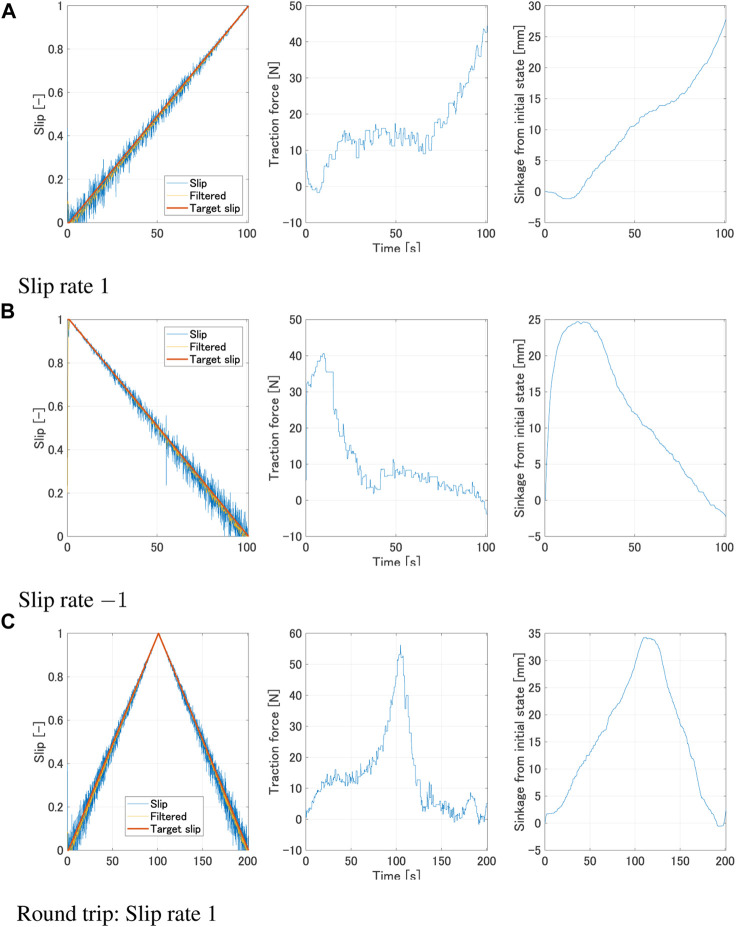
Experimental result with constant slip rate (*ω* = 10°/s, Wheel 1). **(A)** Slip rate 1. **(B)** Slip rate −1. **(C)** Round trip: Slip rate 1.

**FIGURE 3:**
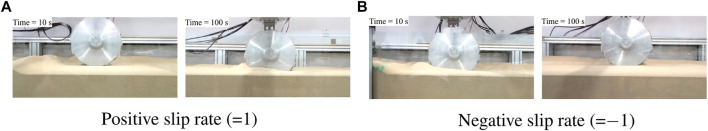
Relationship between wheel sinkage and slip rate. **(A)** Positive slip rate (=1). **(B)** Negative slip rate (=−1).

**FIGURE 4:**
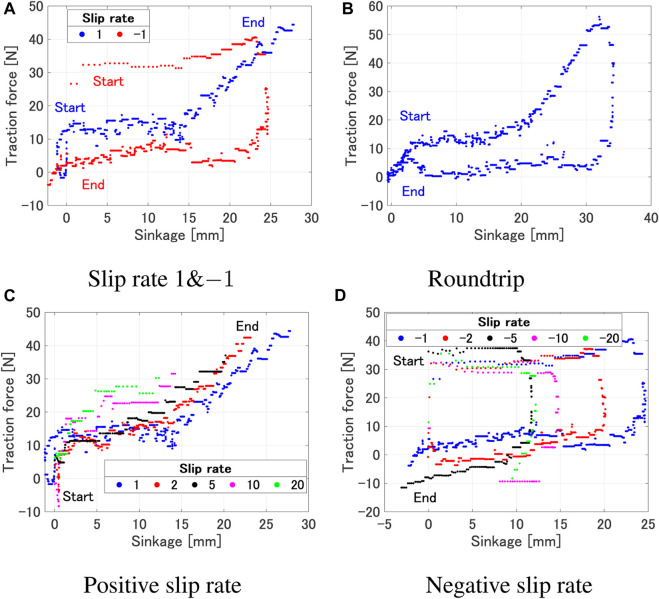
Sinkage vs traction force (*ω* 10°/s, Wheel 1). **(A)** Slip rate 1 and −1. **(B)** Roundtrip. **(C)** Positive slip rate. **(D)** Negative slip rate.

The experiment was designed to observe how the forces acting on the wheel will vary during the transitional phase of increasing/decreasing slip ratio. Futhermore we focused on how the traction force varied with different slip rates. In this study, data acquisition from the single wheel testbed was conducted at a frequency of 10 Hz. The processing of this data involved the use of a moving average filter, specifically implemented with a window size of 5, to ensure optimal data smoothing and accuracy. The traction force gradually increases for a positive slip rate, as shown in Figure 2A. On the other hand, the traction force is seen to gradually decrease for a negative slip rate, as shown in Figure 2B. This can be explained by the amount of wheel sinkage. The traction force is increased as the sinkage increases; the contact area between the wheel and the soil increases (Figure 3A), leading to a larger integrated value of shear stress. On the other hand, when the sinkage decreases, the contact area is reduced (Figure 3B). Therefore, the traction force is reduced due to a smaller integrated value of shear stress. Phenomena where the traction force remains constant despite an increase or decrease in sinkage, can be explained by the normal and shear stresses beneath the wheel. The traction force is obtained by the difference in the longitudinal direction components of normal stress and shear stress. It is believed that the changes in shear stress and normal stress are well-balanced in this region. It can be seen from Figure 2C that the same phenomenon occurs in the round trip experiment as well. Furthermore, even with the same amount of sinkage, the value of the traction force differs depending on the sign of slip rate (Figure 4). Positive slip rates have larger traction force comparing with negative slip rate. This can be explained as follows: the increase in traction force at a positive slip rate is likely due to the amount of sand accumulated on the wheel’s surface. This traction force is a result of the balance between thrust force and traction resistance. With a positive slip rate, sand starts to accumulate in the wheel’s forward direction as the slip ratio approaches 1. In contrast, a negative slip rate leads to the wheel sinking and a large accumulation of sand at the beginning. Even as the slip ratio decreases, more sand is accumulated and pushed forward. The quantity of sand affects traction resistance, suggesting that higher traction forces are generated at a positive slip rate.

**FIGURE 5:**
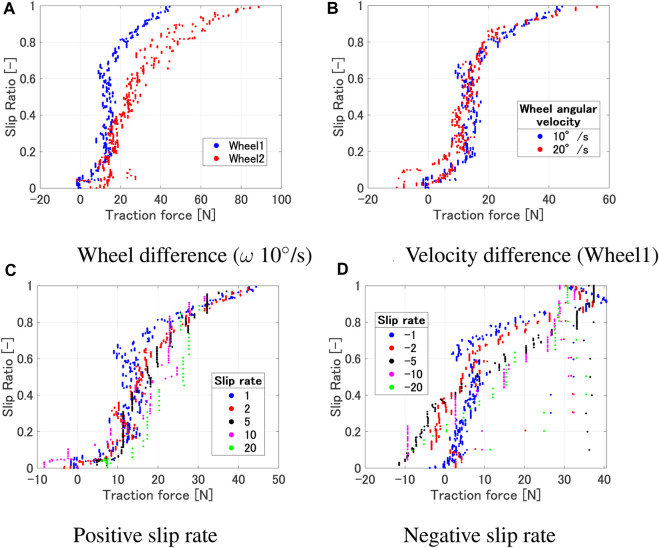
Traction force vs slip ratio. **(A)** Wheel difference (ω 10^◦^/s). **(B)** Velocity difference (Wheel1). **(C)** Positive slip rate. **(D)** Negative slip rate.

Figure 5 shows the relationship of the traction force and slip ratio under various experimental conditions. As shown in Figure 5A, it can be observed that the region of the slip ratio where the traction force remains constant differs depending on the wheel. This is due to the influence of the grouser attached to each wheel. However, from a macro perspective, it can be said that the trend of traction force increasing around a slip ratio of 0–0.2 and saturating between 0.8 and 1 remains consistent. The slip ratio rises with a smaller traction force as the angular velocity increases, but the overall trend where the traction force starts to increase and the saturating region remains consistent (Figure 5B). As shown in Figures 5C, D, the traction force variation where the slip ratio is low or high are similar regardless of the slip rate. However, differences can be observed in the region where the traction force remains constant while the slip ratio increases. When the slip rate is small, a larger slip ratio occurs with a smaller traction force due to a large sinkage. Furthermore, from Figure 4C, even with the same wheel sinkage, a larger traction force occurs when the slip rate is positive and larger. On the other hand, from Figure 5D, it can be observed that a larger traction force occurs when the slip rate is negative and its absolute value is smaller.

In summary, the results indicate that even with the same traction force, the slip ratio varies according to the value of the slip rate. Furthermore, larger traction force occur with positive slip rates than with negative ones, despite the same wheel sinkage. By utilizing these experimentally obtained results in path planning, it becomes possible to generate paths that ensure the rover’s safety and improve its operational efficiency. In the next section, we will discuss the application of the obtained experimental results to path planning.

## 4 Slip rate dependent regression model for path planning

This section describes the path planning method considering slip rate-dependent traversability in the cost function. Modeling slip rate-dependent traversability is realized by elaborating a regression model from the experiment results. Section 4.1 defines the cost function for path planning, and explains how slip ratio could be derived from rover pose and terrain information. Section 4.2 explains the slip rate-dependent regression model to predict slip ratio, and evaluate the fitting accuracy using statistical indicators. Section 4.3 discusses the result obtained from simulation studies.

### 4.1 Cost function for path planning

A path is defined as a series of robot states from the start state to the goal state. The path planner searches for an optimal or sub-optimal path with respect to a specified cost function while satisfying user-defined constraints. In this paper, we introduce a cost function that takes into account of slip rate-dependent traversability. This cost function can be adopted both in graph-based and sampling-based path planning.

First, we consider the balances of forces during slope traversal. From Eq. 3 the transitional acceleration *a* could be derived as follows:

$$a=\frac{dv_{x}}{dt}=-r\omega \dot{s}$$ (5)

The traction load *F*_*tr*_ acting on the rover balances with the traction force *F*_*x*_, which is the sum of wheel traction forces $F_{x}=\sum F_{x_{i}}$ . Thus the equation of motion can be described as follows:

$$m\ddot{x}=F_{x}+mg\sin\theta_{y}$$ (6)

where *m* is the mass of the rover, *θ*_*y*_ is the rover pitch angle, and *g* is the gravity. The pitch angle will be positive when descending a slope, and negative when ascending. The traction force could be derived by substituting Eq. 5, 6.

$$-\left(mr\omega \dot{s}+mg\sin\theta_{y}\right)=F_{x}$$ (7)

The traction force can be expressed as a function $F_{x}\left(\theta_{y},\dot{s}\right)$ with pitch angle and slip rate as inputs. The slip ratio can be estimated from the relationship between the traction force and slip ratio obtained from the experimental results. Therefore, the slip ratio estimation model is expressed as $s\left(F_{x}\left(\theta_{y},\dot{s}\right)\right)=s\left(\theta_{y},\dot{s}\right)$ . In this case, the slip rate is treated as a parameter. If the slope angle increases, the slip rate will take a positive value, and if the slope angle decreases, it will take a negative value. As a result, to consider the slip variation we defined the cost between adjacent nodes *c*_*ij*_ as follows:

$$c_{ij}=W_{s}\frac{\left|s\left(\theta_{y},\dot{s}\right)\right|}{N_{s}}+W_{\theta_{x}}\frac{\left|\theta_{x}\right|}{N_{\theta_{x}}}$$ (8)

where *θ*_*x*_ is the rover roll angle, *W*_•_ are weighting factors, *N*_•_ is the normalization factor. Here nodes refer to either nodes utilized in graph search planning or sampling states in sampling-based planning. The definition of nodes used in this paper will be mentioned later. The relationship between the traction force and slip ratio obtained from the experimental results is made more manageable by creating a regression model. Details about the regression model will be discussed in the next subsection. The rover roll angle is introduced to minimize risks such as rollover. Calculating the roll and pitch angle at each node would be sufficient for the cost function. Slip rate-dependent traversability can be defined as the slip ratio term included in the cost function. Incorporating changes in the slip ratio based on slope variations, namely, the slip rate, our approach effectively tackles quasi-dynamic conditions.

**FIGURE 6:**
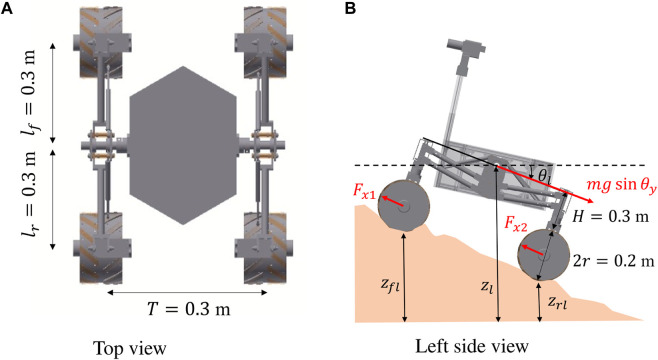
Schematic view of rover. **(A)** Top view. **(B)** Left side view.

The rover considered in this paper is assumed to be a 4 wheeled mobile robot with a differential suspension. The rover mass is 38.5 kg and each dimension is shown in Figure 6. The rover roll and pitch angle are calculated based on the geometrical constrain between the differential suspension mechanism and the terrain surface. The height of each wheel *z*_fl_, *z*_rl_, *z*_rr_, and *z*_fr_ is derived by a DEM (Digital Elevation Model) node surrounding each wheel’s contact point. From the wheel height the left joint angle *θ*_*l*_ and the right joint angle *θ*_*r*_ can be derived as below:

$$\theta_{\text{l}}=\arcsin\left(\frac{z_{\text{rl}}-z_{\text{fl}}}{l_{\text{f}}+l_{\text{r}}}\right)$$ (9)

$$\theta_{r}=\arcsin\left(\frac{z_{\text{rr}}-z_{\text{fr}}}{l_{\text{f}}+l_{\text{r}}}\right)$$ (10)

where *l*_f_ and *l*_r_ are the length from the joint to the front wheel and rear wheel, respectively. The height of the left joint angle *z*_l_ and the right joint angle *z*_r_ can be calculated based on the wheel contact points and geometric constraints of the rover:

$$z_{\text{l}}=z_{\text{rl}}+\left(H+2r\right)\cos\theta_{\text{l}}-l_{r}\sin\theta_{\text{l}}$$ (11)

$$z_{\text{r}}=z_{\text{rr}}+\left(H+2r\right)\cos\theta_{\text{r}}-l_{\text{r}}\sin\theta_{\text{r}}$$ (12)

where *H* is the height from the wheel to the body, and *r* is the wheel radius. Finally, the roll *θ*_*x*_ and pitch *θ*_*y*_ angles are geometrically calculated as below:

$$\theta_{x}=\arcsin\left(\frac{z_{\text{l}}-z_{\text{r}}}{T}\right)$$ (13)

$$\theta_{y}=\frac{\theta_{\text{l}}+\theta_{\text{r}}}{2}$$ (14)

where *T* is the distance between the left and right wheels.

### 4.2 Regression model

To consider slip rate-dependent traversability in the cost function for path planning, we create a regression model between the traction force and the slip ratio. The traction force is calculated from the difference between thrust force and traction resistance, and the relationship between thrust force and traction resistance changes as the slip ratio varies. Theoretically, a traction force does not occur unless the wheel slips to a certain extent, and around a slip ratio of 1 (a stuck state), the traction force saturates. Therefore, we chose a regression model based on the sigmoid function and represented it by the following equation:

$$s\left(F_{x}\right)=k_{1}+\frac{k_{2}}{1+e^{k_{3}\left(F_{x}-k_{4}\right)}}$$ (15)

where *k*_1_, *k*_2_, *k*_3_, *k*_4_ are coefficients. Here, *k*2/*k*1 represents the scaling of the slip ratio, and *k*4/*k*3 indicates the offset amount of the traction force. Other regression functions were considered too, and the results are shown in the Appendix A. From the experimental results for each slip rate, we calculated the coefficients for Eq. 15 to check how well the regression curve fits. By iteratively adjusting the parameters of a chosen nonlinear model to minimize the difference between observed data and the model’s predictions. Levenberg-Marquardt was used for the optimization algorithm. The metrics used in this study are the Sum of Squares due to Error (SSE), R-square, Degrees of Freedom for error (DFE), Adjusted R-square, and Root Mean Squared Error (RMSE). SSE measures the total deviation of the response values from the fit to the response values. R-square is the square of the correlation between the response values and the predicted response values. R-square can take on any value between 0 and 1, with a value closer to 1 indicating that a greater proportion of variance is accounted for by the model. DFE is an adjustment of the R-square statistic previously defined, based on the residual degrees of freedom. The adjusted R-square statistic is generally the best indicator of the fit quality when you compare. The adjusted R-square statistic can take on any value less than or equal to 1, with a value closer to 1 indicating a better fit. RMSE is an estimate of the standard deviation of the random component in the data.

**FIGURE 7:**
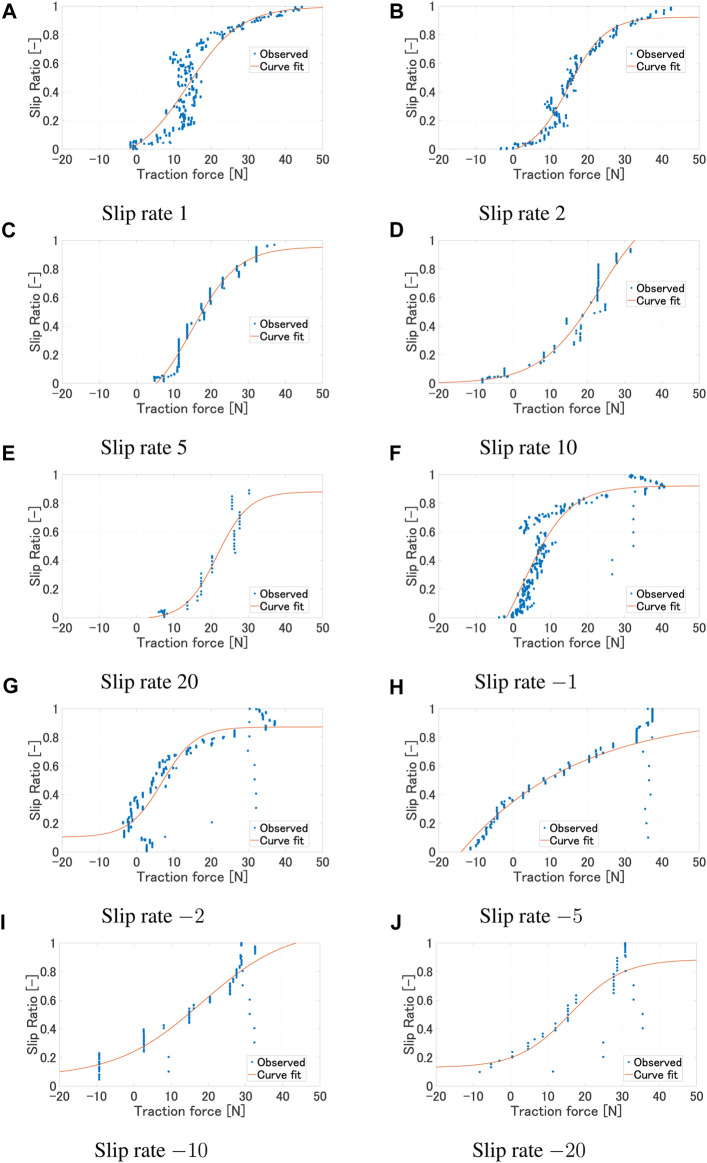
Regression model for each slip rate (*ω* 10°/s, Wheel 1). **(A)** Slip rate 1. **(B)** Slip rate 2. **(C)** Slip rate 5. **(D)** Slip rate 10. **(E)** Slip rate 20. **(F)** Slip rate −1. **(G)** Slip rate −2. **(H)** Slip rate −5. **(I)** Slip rate −10. **(J)** Slip rate −20.

**FIGURE 8:**
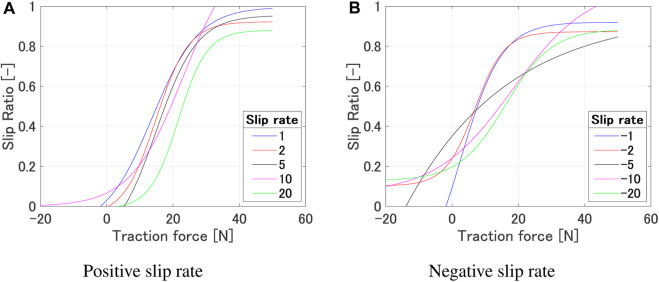
Relationship between each regression model (*ω* 10°/s, Wheel 1). **(A)** Positive slip rate. **(B)** Negative slip rate.

Figure 7 shows the experimental results and the regression model for each slip rate. Figure 8 shows the relationship between each regression model. Table 1 shows the values of each metric when the regression curve is applied. Table 2 shows the coefficients of the regression curve. Focusing on the adjusted R-square, we can see that it shows values above 0.8 for results with absolute slip rates of 2, 5, and 10, indicating a good fit. On the other hand, the fitting accuracy decreases for absolute slip rates of 1 and 20. We believe that the decrease in fitting accuracy for an absolute slip rate of 1 can be attributed to a region where the slip rate increases vertically. Furthermore, for an absolute slip rate of 20, we believe the reduced fitting accuracy is due to the shorter time it takes to reach a slip ratio of 1 (or 0), resulting in fewer data points to represent the relationship between traction force and slip ratio. The statistical analysis of the regression model confirms that the model can estimate the slip ratio within an accuracy of 85% in average. The data presented in Table 2 indicate that the coefficient exhibits a range of values across different slip rates. This variation suggests the impracticality of applying a uniform coefficient for all slip rates, highlighting the need for a more nuanced approach in coefficient selection. However, since the primary focus is on path planning using the estimated slip rate values for a given traction force and their variations, further modeling of the slip rate and its validity verification is out of scope.

**TABLE 1:** Statistics for curve fitting (Sigmoid function).

Slip rate	SSE	R-Square	DFE	Adjusted R-square	RMSE
1	2.01e+05	0.757	937	0.756	14.6
2	2.13e+04	0.950	476	0.950	6.69
5	4.71e+03	0.973	192	0.973	4.95
10	6.77e+03	0.928	100	0.926	8.23
20	3.83e+03	0.924	54	0.920	8.42
−1	1.98e+05	0.757	940	0.756	14.5
−2	7.98e+04	0.809	475	0.808	13.0
−5	2.45e+04	0.857	195	0.855	11.2
−10	1.38e+04	0.839	98	0.834	11.9
−20	1.29e+04	0.712	52	0.695	15.7

**TABLE 2:** Sigmoid function coefficients.

Slip rate	*k*_1_	*k*_2_	*k*_3_	*k*_4_
1	99.64	−110.6	0.1409	13.72
2	−5.068	97.38	−0.2022	14.87
5	95.51	−115.4	0.162	14.95
10	130.5	−130.6	0.1254	23.17
20	87.99	−89.23	0.2327	21.63
−1	−44.19	136.3	−0.1616	2.664
−2	10.26	77.04	−0.2235	6.863
−5	−2,824	2,921	−0.03337	−115.1
−10	6.282	104.4	−0.08599	18.31
−20	12.87	75.73	−0.1426	16.14

For each slip rate, the possible traction force is calculated using Eq. 6, and the slip ratio is estimated using the generated regression model based on the calculated results. Since the slip ratio estimated for each slip rate varies, the mean, standard deviation, maximum, and minimum values are computed. For this study, the slip ratio used in Eq. 8 will be based on the mean value.

### 4.3 Path planning simulation

To verify the usefulness of the proposed slip rate-based traction force - slip ratio regression model, we conducted path planning simulations for crater type environments and rough terrain environments. Each terrain has an uneven height information generated by the fractal approach (Yokokohji et al., 2004) to simulate planetary surface roughness. We use DEM as an expression for the terrain data, and by treating each grid point as a node, we execute path planning. In this study, we decided to adopt the A* algorithm (Hart et al., 1968) for path planning and defined the cost function between nodes as $f_{proposed_{ij}}=c_{ij}+g_{ij}$ *g*_*ij*_ is a heuristic function, and in this paper, it is defined as the Euclidean distance from the next node where the rover will move to the goal. Additionally, as a comparison for the proposed method, we define the cost function as below:

$$f_{classical_{ij}}=W_{\theta_{x}}\frac{\theta_{x}}{N_{\theta_{x}}}+W_{\theta_{y}}\frac{\theta_{y}}{N_{\theta_{y}}}+g_{ij}$$ (16)

where *θ*_*x*_ is the rover roll angle, *θ*_*y*_ is the rover pitch angle, *W*_•_ are weighting factors, *N*_•_ is the normalization factor. We use the same heuristic function as the proposed method. Thresholds were set for the roll and pitch angles, ensuring that paths where the roll or pitch angle exceeds the threshold are not generated. The normalization factor for the roll and pitch angles was set to be the same as this threshold value. The weight factor for both was set to 0.5, the threshold for each angle were set to 10.0°, and the wheel angular velocity was set to 10°/s to employ the regression model. Futhermore, to observe the effect of the weighting factor, simulations were conducted with different types of *W*_*s*_ for the proposed path planning: Incremental values ranging from 0.1 to 0.9, with steps of 0.1, were used for analysis. The sum of the weighting factors is assumed to be 1; in other words, $W_{\theta_{x}}$ is taken to be 1 − *W*_*s*_. Path tracking employing a dynamics simulator was not implemented in this study due to its close association with control theory and the potential variability in the rover’s slip ratio based on the design of the control method. Instead, the results focused on evaluating the generated path by analyzing the shape and the estimated slip ratio obtained from Eq. 15.

**FIGURE 9:**
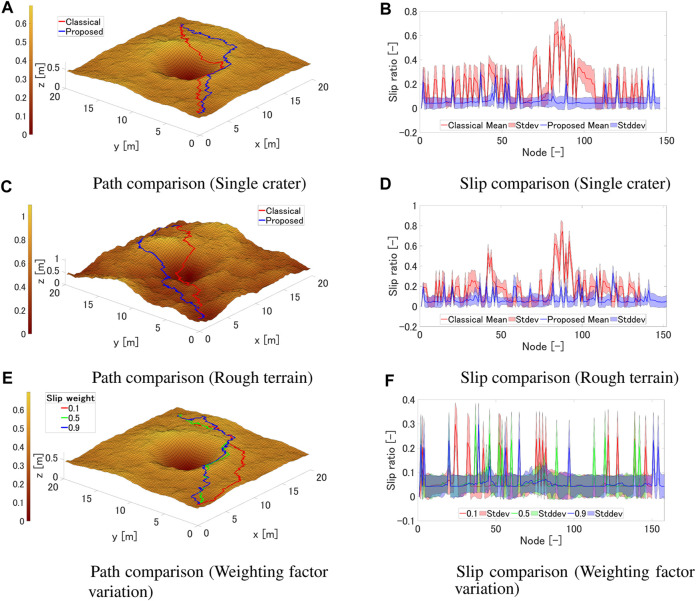
Path planning results. **(A)** Path comparison (Single crater). **(B)** Slip comparison (Single crater). **(C)** Path comparison (Rough terrain). **(D)** Slip comparison (Rough terrain). **(E)** Path comparison (Weighting factor variation). **(F)** Slip comparison (Weighting factor variation).

Figure 9 shows the results of generated paths for two representative terrains. The start node is (*x*, *y*) = (1.0, 1.0) and the goal node is (*x*, *y*) = (18.0, 18.0). As can be seen from Figure 9A, the classical method generates a path that passes close to the inner rim of the crater, while the proposed method generates a path that slightly avoids the rim of the crater. This can be explained from Figure 9B, showing that the proposed path is passing through regions with a smaller slip ratio. Throughout the journey, the expected mean slip ratio is 0.189, with a expected maximum slip ratio of 0.753, and a standard deviation of 0.151 for the classical path. For the proposed path the expected mean slip ratio is 0.0633, with a expected maximum slip ratio of 0.439, and a standard deviation of 0.0459. As can be seen from Figure 9C, both the classical and proposed methods produce similar paths. Near the center of the terrain at (*x*, *y*) = (10.0, 10.0), the classical method overcomes a steep slope, whereas the proposed method travels relatively small inclination slopes. This can be inferred to be due to the proposed method generating a path that results in a smaller slip ratio, which can also be explained from Figure 9D; showing that the proposed path is passing through regions with a smaller slip ratio. Throughout the journey, the expected mean slip ratio is 0.178, with a expected maximum slip ratio of 0.845 for the classical path. For the proposed path the expected mean slip ratio is 0.0747, with a expected maximum slip ratio of 0.459.

Figure 9E shows the results when the weighting factor is changed for a single crater environment. From Figure 9E, it can be seen that by changing the weight for the slip ratio term, the shape of the generated path varies significantly. For example, when the slip ratio is hardly considered, where *W*_*s*_ = 0.1, paths are generated that involve traveling far away from the crater rim and occasionally passing through bumps, causing large slip to occur. On the other hand, when considering the slip ratio term, it is possible to reduce the number of occurrence for large slips. From Table 3, it can be observed that by increasing the weighting factor, the mean slip and the maximum slip could be decreased. Especially, when *W*_*s*_ exceeds 0.7, the mean value of slip ratio tends to be reduced. From Table 3, it can be observed that the mean value of slip ratio tends to be reduced when *W*_*s*_ exceeds 0.5. These results indicate that the boundaries of the weighting factors are determined based on the terrain, and it becomes evident that there are differences in the slip ratios that occur on either side of these boundaries. Further examination should be taken into account with various terrain conditions.

**TABLE 3:** Effect of weighting factor for slip ratio (Single crater).

*W*_*s*_	Single crater			Rough terrain		
	Mean slip [-]	Max slip [-]	Std. dev [-]	Mean slip [-]	Max slip [-]	Std. dev [-]
0.1	0.0666	0.299	0.0588	0.0950	0.498	0.0804
0.2	0.0639	0.289	0.0498	0.0815	0.348	0.0658
0.3	0.0596	0.281	0.0508	0.0823	0.348	0.0690
0.4	0.0621	0.299	0.0460	0.0823	0.348	0.0648
0.5	0.0633	0.297	0.0515	0.0747	0.329	0.0582
0.6	0.0660	0.341	0.0599	0.0739	0.338	0.0555
0.7	0.0580	0.228	0.0419	0.0764	0.338	0.0544
0.8	0.0600	0.336	0.0460	0.0750	0.328	0.0544
0.9	0.0590	0.297	0.0427	0.0747	0.329	0.0582

From the above results, the proposed method is possible to generate a path that can reduce the expected slip ratio. Especially in the example of crater traversal, a reduction of 66% in the slip ratio was confirmed, and in the example of rough terrain traversal, a reduction of 58% was confirmed. Also, we confirmed that by increasing the weighting factor of the slip ratio term, it is possible to suppress potential slips.

### 4.4 Dynamics simulation

Dynamics simulators are pivotal in the field of robotics research, where they are extensively used for testing and proving theoretical methods. Given that robots can be expensive, fragile, and scarce, it is a common practice to initially or exclusively conduct tests and validations within these simulators. The development of a wide range of dynamics simulators has significantly facilitated this process, enabling researchers to explore and refine their ideas in a controlled and cost-effective virtual environment (Collins et al., 2021).

Project Chrono (Mazhar et al., 2013), an open-source physics engine, is particularly reasonable for simulating sandy terrains. Its capability to model granular materials such as sand is crucial for understanding the challenges unique to sandy environments, like shifting surfaces and variable traction. The engine provides high-fidelity simulations of complex physical interactions, including the dynamics of friction, slippage, and terrain deformation, essential for realistic representation of wheel-soil interactions. The Soil contact model in Project Chrono is a sophisticated simulation tool based on terramechanics principles. It leverages the Bekker theory, a foundational framework in terramechanics, to understand and calculate the pressure-sinkage relationships and shear displacement in deformable terrains. Soil contact model allows for customization of soil parameters such as cohesion, internal friction angle, and compaction properties, enabling precise simulations of different soil types. The model’s capability to simulate complex interactions between wheel load, motion, and soil characteristics, along with its integration into the multi-body dynamics makes it an invaluable resource for comprehensive studies of robot dynamics. From the above mentioned features, Project Chrono is chosen as the dynamics simulator for this study.

The soil parameters are set to match the experimental conditions. The robot configuration is the same as the one used in path planing simulation. Each simulation step was set to 0.001 s, the rover wheel angular velocity to 20°/s, and the path tracking algorithm was implemented by employing the slip compensation method (Ishigami et al., 2009). The slip rate in the dynamics simulation can be derived by dividing the slip ratio by the time step.

**FIGURE 10:**
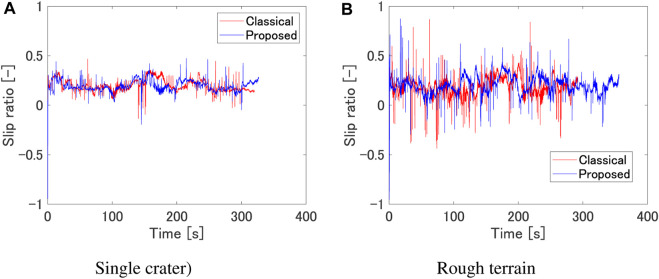
Dynamics simulation results. **(A)** Single crater. **(B)** Rough terrain.

Figure 10 show the results of dynamics simulation for two representative terrains. From Figure 10A, it can be seen that the rover’s slip ratio is reduced by using the proposed method where the rover travels near the crater rim. The mean value of slip ratio is 0.201 for the classical path, and 0.197 for the proposed path. The mean value of slip rate is 0.606 for the classical path, and 0.591 for the proposed path. These results insist that the proposed method can reduce the slip ratio and slip rate. From Figure 10B, it can be seen that the rover’s slip ratio does not change significantly by using the proposed method; however, the slip rate is reduced. The mean value of slip ratio is 0.188 for the classical path, and 0.205 for the proposed path. The mean value of slip rate us 0.898 for the classical path, and 0.674 for the proposed path. The decrease in slip rate is 2% in the single crater scenario, and 25% for the rough terrain scenario. This suggests that the use of the proposed cost results in the generation of paths with minimal variations in slope gradient. As observed in Figure 9C, it is also evident that the proposed method avoids regions with significant undulations, particularly in the central area. We have also conducted dynamics simulation for the weighting factor variation; Table 4 shows the mean value of slip ratio and slip rate for each weighting factor. As the weighting factor for the slip term increases, it is observed that the slip ratio does not change significantly, while the slip rate tends to decrease.

**TABLE 4:** Dynamics simulation results with different weighting factor (Rough terrain).

	Mean slip ratio [-]	Slip ratio std. dev [-]	Mean slip rate [1/s]	Slip rate std. dev [-]
0.1	0.189	0.0765	0.847	1.75
0.2	0.196	0.0764	0.771	1.61
0.3	0.197	0.0840	0.723	1.45
0.4	0.196	0.0811	0.863	1.71
0.5	0.205	0.0840	0.674	1.27
0.6	0.211	0.0936	0.697	1.57
0.7	0.206	0.0845	0.683	1.42
0.8	0.192	0.0799	0.640	1.48
0.9	0.191	0.0832	0.639	1.45

In the real world, it is observed that the slip ratio does not change stepwise, indicating that simulators may not adequately represent transitional states. This underscores the necessity for further verification through dynamics simulations that incorporate wheel-soil interaction during transitional states, as well as experiments conducted on rough terrain.

## 5 Conclusion and future work

We focused on the transient slip, or slip rate the time derivative of slip ratio, to explicitly address it into the cost function of path planning algorithm. First, we defined the term slip rate and conducted single wheel testbed experiments with a constant slip rate. The experimental results revealed that even with the same traction force, the possible range of the slip ratio depends on the slip rate. Regarding the experimental results, we applied a regression model to the relationship between traction force and slip ratio taking into account of slip rate. For each regression model, we evaluated the fitting accuracy using statistical indicators. As a result, we confirmed that a regression model based on the sigmoid function is suitable, with an average Adjusted R-square of 0.847. Lastly, we proposed a cost function employing the generated regression model for path planning, and confirmed that it is possible to minimize the slip ratio experienced by the rover when driving near craters by simulation studies. Especially in the example of crater traversal, a reduction of 66% in the slip ratio was confirmed, and in the example of rough terrain traversal, a reduction of 58% was confirmed in the path planning simulation. The dynamics simulation results also confirmed that the proposed method can reduce the slip rate in each terrain.

A possible future direction of this study is efficient data collection to improve the accuracy of the slip rate experiment. The experimental conditions assumed a small rover, with the wheel’s angular speed being relatively slow and the wheel load being light. By increasing these conditions and enriching the data, it is possible to create a more versatile regression model. Moreover, not only conducting experiments on flat terrain, but it is also meaningful to collect data under conditions of climbing or descending slopes.

Another future work possibly includes the implementation of a new path tracking method based on the experimental results we observed in this paper. It is acknowledged that there is a need to develop operation plans that minimize the slip ratio by integrating wheel-soil interaction to both path planning and path tracking. Futhermore an online path planning method can be implemented in the path planning phase. Using online path planning methods, it is possible to compute the optimal path considering the changes in the rover’s slip ratio. Employing the regression model, based on the insights obtained from the experimental results, it is possible to regenerate a path that is safe and has a high travel efficiency.

## Data Availability

The raw data supporting the conclusion of this article will be made available by the authors, without undue reservation.

## Appendix A

In addition to the functions discussed in Section 3.2, further curve fitting analysis were conducted. For this analysis, functions frequently utilized in the field of curve fitting were selected as the basic functions. The primary functions employed in this study include the Hill function, Boltzmann function, five-parameter sigmoid, logistic function, and polynomial. Each of these functions is expressed as follows:

$$s\left(F_{x}\right)=k_{1}\frac{{F_{x}}^{k_{2}}}{{k_{3}}^{k_{2}}+{F_{x}}^{k_{2}}}$$
(17)

$$s\left(F_{x}\right)=\frac{k_{1}-k_{2}}{1+e^{\left(F_{x}-k_{3}\right)/k_{4}}}+k_{2}$$
(18)

$$s\left(F_{x}\right)=k_{1}+\frac{k_{2}}{{\left[1+e^{-\left(\frac{F_{x}-k_{3}}{k_{4}}\right)}\right]}^{k_{5}}}$$
(19)

$$s\left(F_{x}\right)=\frac{k_{1}}{1+e^{-k_{2}\left(F_{x}-k_{3}\right)}}$$
(20)

$$s\left(F_{x}\right)=\overset{n}{\underset{i=0}{\sum }}k_{i}{F_{x}}^{i}$$
(21)

where *k*_*i*_ are the coefficients. The fitting accuracy of each function was evaluated using the same statistical indicators as the sigmoid function, and is shown in Table 5. It can be seen that the polynomial function (ninth order) has the highest fitting accuracy; however there might be a risk of overfitting. Other functions have similar fitting accuracy to the proposed sigmoid function, and it can be concluded that the proposed function could be suitable for the regression model. To further enhance the reliability of our curve fitting approach, additional data acquisition and a fundamental understanding of the underlying physical phenomena are required.

**TABLE 5:** Statistics for curve fitting (Different regression model).

Slip rate	SSE	R-Square	DFE	Adjusted R-square	RMSE
Proposed	20.07	0.757	937	0.756	0.146
Hill function	20.31	0.714	886	0.7138	0.151
Boltzmann function	20.07	0.757	937	0.756	0.146
Five Parameter Sigmoid	19.95	0.758	936	0.757	0.146
Sigmoid Logistic	20.27	0.754	938	0.7537	0.147
Polynomial	19.07	0.769	931	0.767	0.143

There is another approach to use learning method based regression. For example, Gaussian Process Regression (GPR), a non-parametric, Bayesian approach to regression, offers a flexible and robust framework for modeling complex data. At its core, GPR operates under the assumption that the observed data can be represented as a realization of a Gaussian process; a collection of random variables, any finite number of which have a joint Gaussian distribution. The Squared Exponential Kernel was used to conduct the GPR based regression. By using the GPR the regression model can be expressed in 0.846 of Adjusted R-square, which exceeds the proposed sigmoid function based regression models. GPR can offer flexibility and robust uncertainty quantification, but can be less intuitive for physical interpretation due to its non-parametric nature and complexity of the kernel function.
